# Supplementary material for: The crystal structure of Cry78Aa from Bacillus thuringiensis provides insights into its insecticidal activity
Source: Commun Biol. 2022 Aug 9;5:801. doi: 10.1038/s42003-022-03754-6 (PMC9363482; doi:10.1038/s42003-022-03754-6)
Supplement: Supplementary file 2 — Supplementary information [file 42003_2022_3754_MOESM2_ESM.pdf]

Supplementary Figure 1. Phylogenetic tree analysis of 311 model Cry proteins.

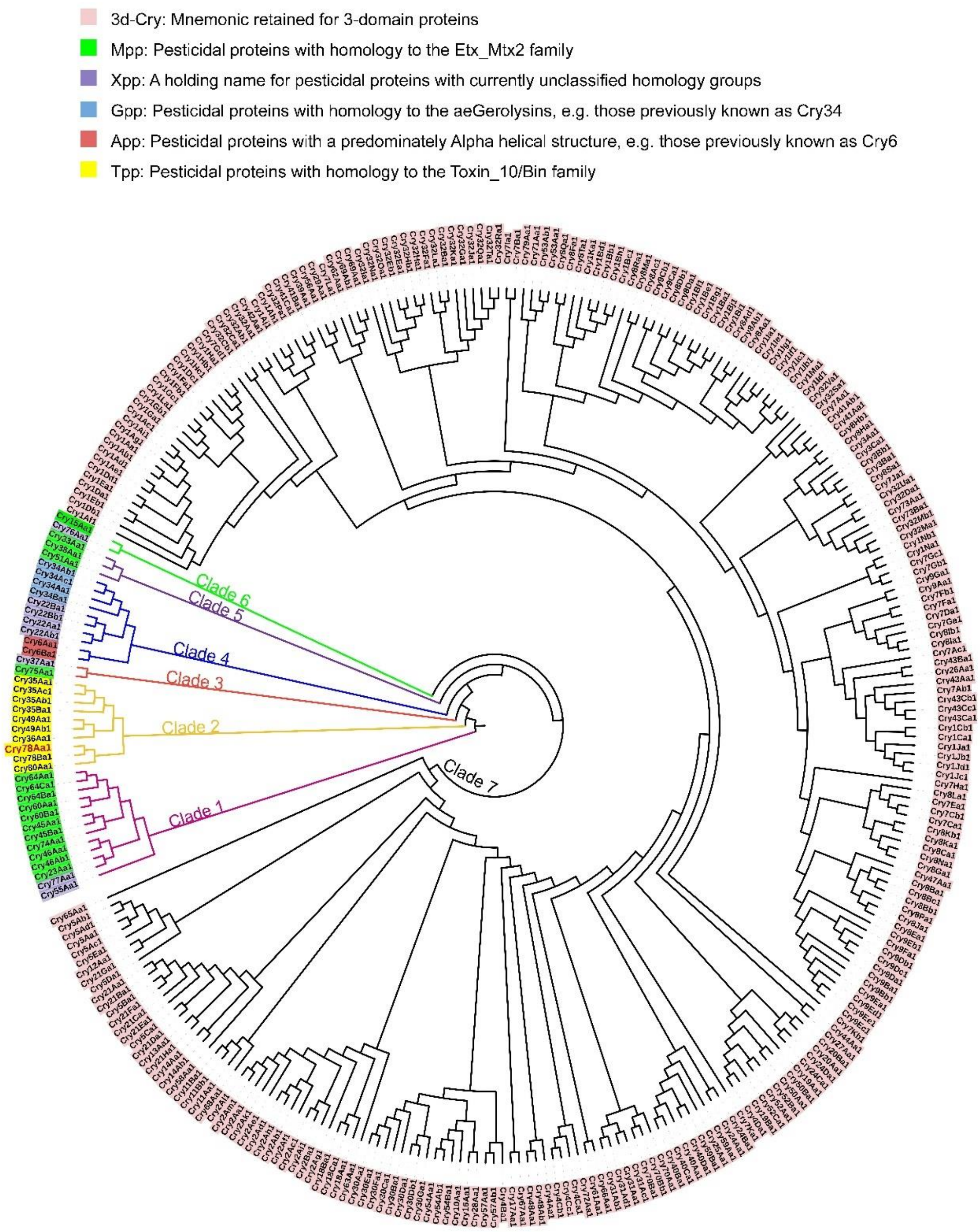

According to the information published by the pesticide protein database of the Bacterial Pesticide Protein Resource Center (https://www.bpprc.org/), a total of 311 amino acid sequences of model Cry proteins were downloaded, such as Cry1Aa1, Cry2Ab1, Cry78Aa1. The phylogenetic tree of these sequences was constructed by MEGA X (10.0.2) using the unweighted pair-group method with arithmetic means (UPGMA), which was displayed and annotated by using the website of Interactive Tree of Life (https://itol.embl.de/). Different clades are shown with colored branches. The Cry78Aa protein is shown with a red label.

Supplementary Figure 2. A stable band of Cry78Aa formed after digestion by limited proteolysis.

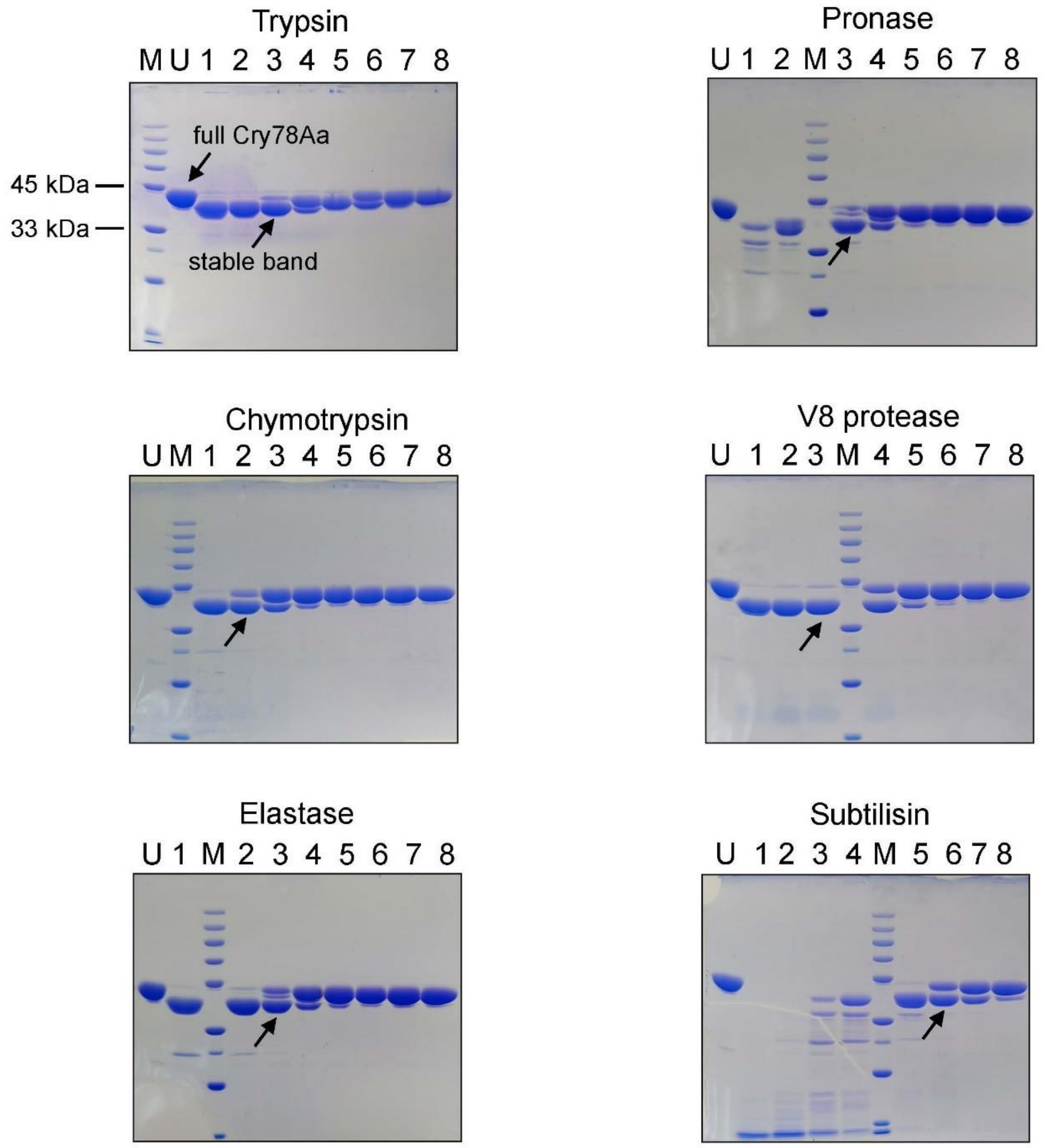

Full Cry78Aa was digested by six kinds of protease. A fixed amount of protein (0.1 mg ml^{-1}) was incubated with eight gradient concentrations of protease for 30 minutes, followed by phenylmethylsulfonyl fluoride (PMSF) addition and boiling to end the digestion reaction. The samples were then loaded for SDS–PAGE and Coomassie brilliant blue staining. Downward arrows floating on the picture represent undigested full-length Cry78Aa, and the upward arrows represent the stable band which may have resulted from the removal of several amino acids from full-length Cry78Aa. M represents the protein marker, and U represents undigested Cry78Aa. Numbers 1 to 8 represent a decreased concentration slope of the protease used. The molecular weight of the specific band in the marker is indicated as a reference.

Supplementary Figure 3. Comparisons of Cry78Aa with its structural homologs.

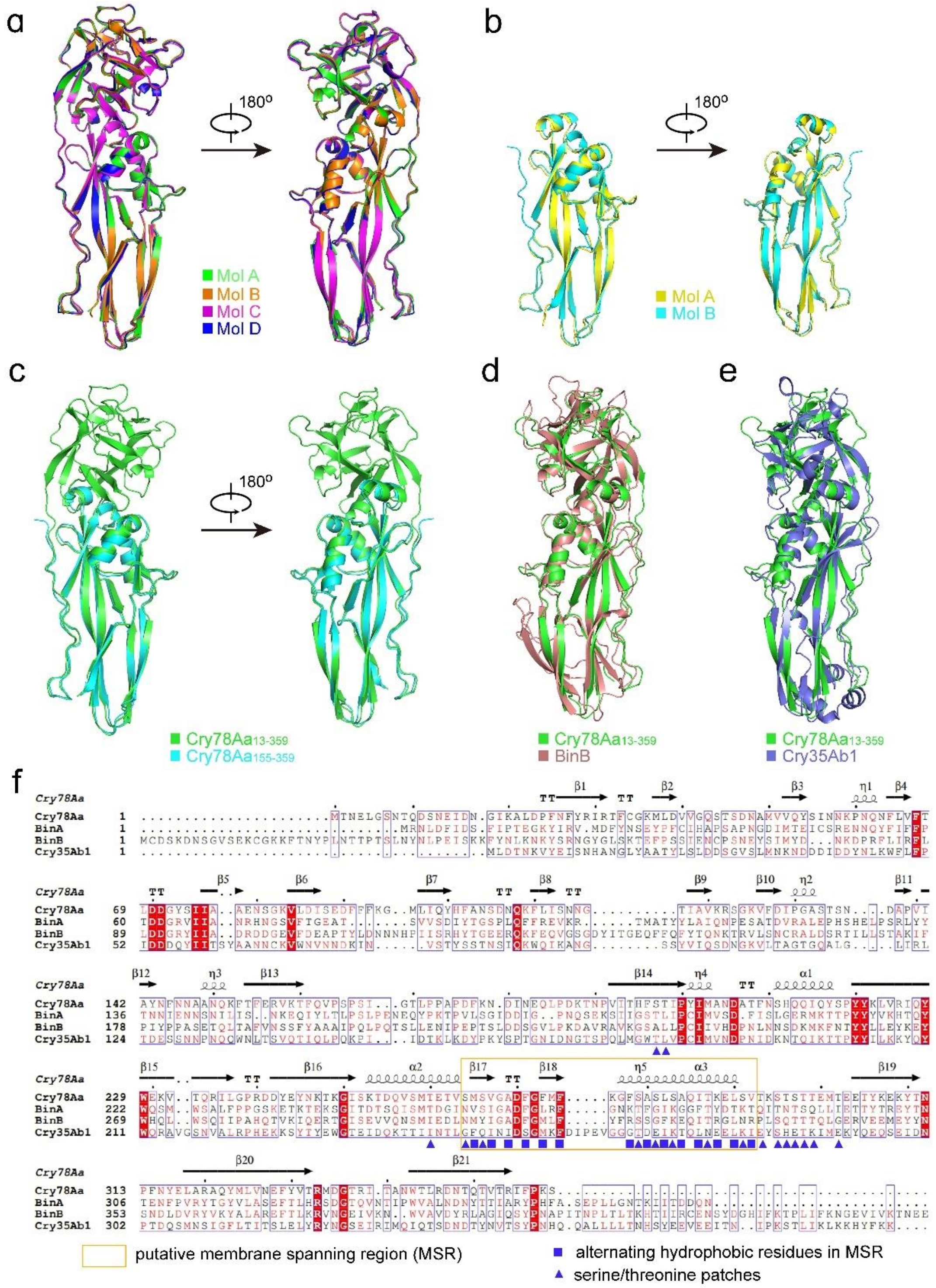

(a) Structural alignment of the four molecules in the crystal structure of Cry78Aa$_{13\text{-}359}$. Four protomers nearly completely superimposed. **(b)** Structural alignment of the two molecules in the crystal structure of Cry78Aa$_{155\text{-}359}$. Two protomers nearly completely superimposed. **(c)** Structure alignment of Cry78Aa$_{13\text{-}359}$ (Mol A) and Cry78Aa$_{155\text{-}359}$ (Mol B). The two structures were highly identical, implying that the pore-forming domain of Cry78Aa did not undergo any conformational change in the presence of the individual NTDs. **(d)** Structure alignment of Cry78Aa$_{13\text{-}359}$ (Mol A) with Bin B (PDB:

5FOY, Mol B). The two structures were overall similar to each other, and the pore-forming domains superposed better than their trefoil domains. **(e)** Structure alignment of Cry78Aa$_{13-359}$ (Mol A) with Cry35Ab1 (PDB: 4JP0). The results of the comparison were similar to those in diagram (d). **(f)** Sequence alignment of Cry78Aa with its structural homologs. Secondary structural units of Cry78Aa$_{13-359}$ are shown above. The numbers to the side specify the start and end of each sequence.

Supplementary Figure 4. Interaction of the NTD of Cry78Aa with its CTD.

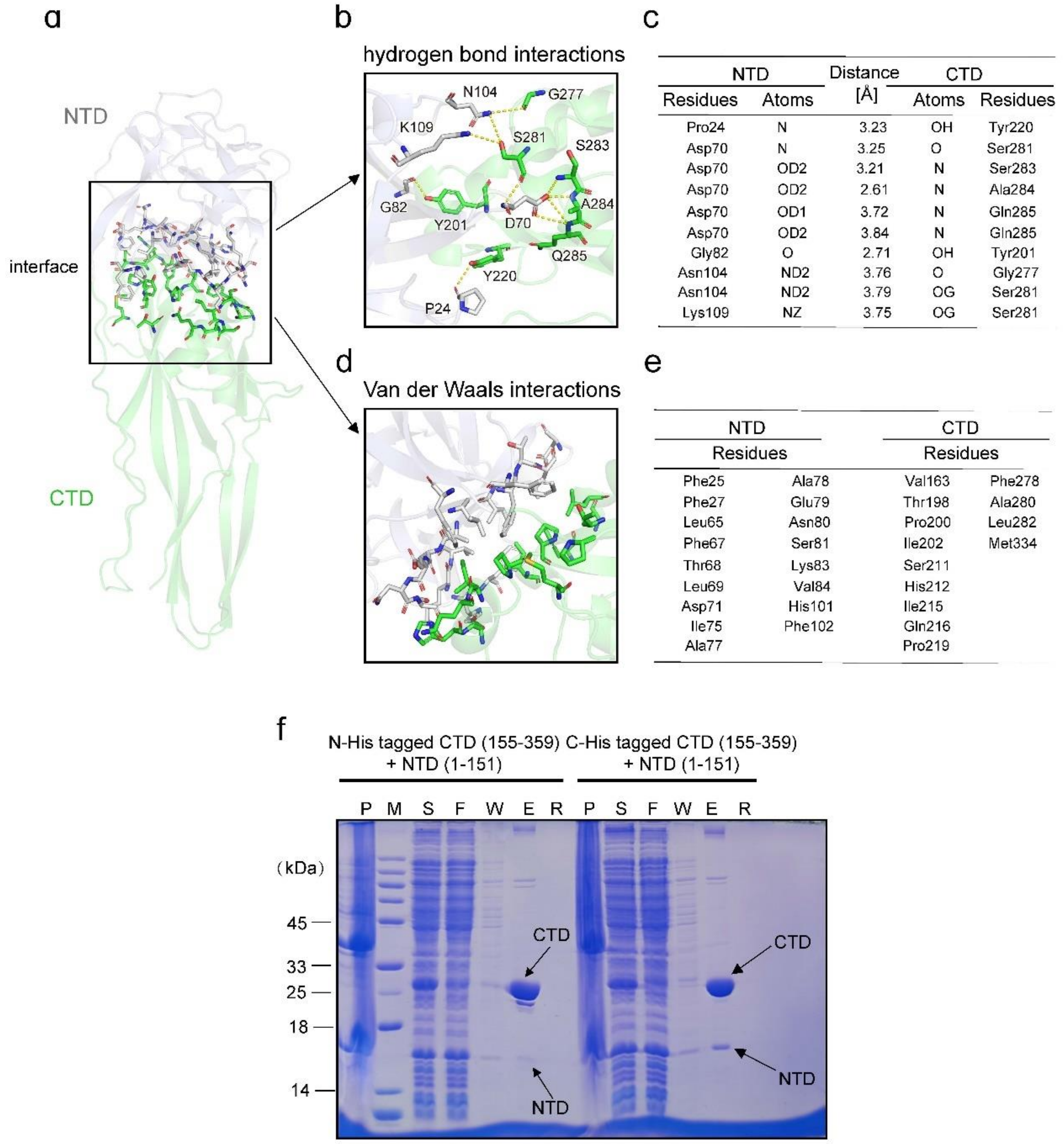

NTD Residues	NTD Atoms	Distance [Å]	CTD Atoms	CTD Residues
Pro24	N	3.23	OH	Tyr220
Asp70	N	3.25	O	Ser281
Asp70	OD2	3.21	N	Ser283
Asp70	OD2	2.61	N	Ala284
Asp70	OD1	3.72	N	Gln285
Asp70	OD2	3.84	N	Gln285
Gly82	O	2.71	OH	Tyr201
Asn104	ND2	3.76	O	Gly277
Asn104	ND2	3.79	OG	Ser281
Lys109	NZ	3.75	OG	Ser281

NTD Residues		CTD Residues	
Phe25	Ala78	Val163	Phe278
Phe27	Glu79	Thr198	Ala280
Leu65	Asn80	Pro200	Leu282
Phe67	Ser81	Ile202	Met334
Thr68	Lys83	Ser211	
Leu69	Val84	His212	
Asp71	His101	Ile215	
Ile75	Phe102	Gln216	
Ala77		Pro219	

(a) The interface of the NTD and CTD of Cry78Aa. The NTD and CTD are shown in light blue and green, respectively. The residues that may participate in the interaction of the NTD and CTD are enclosed in a frame and shown as sticks (color representations: carbon, light blue for the NTD and blue for the CTD; nitrogen, blue; oxygen, red; sulfur, yellow). The ribbon representation of other residues is shown with partial transparency for clarity. **(b)** Close-up view of the residues that participate in hydrogen bond interactions of the NTD and CTD of Cry78Aa. The residues are shown as sticks, and their categories and numbers are labeled. The yellow dashed lines represent hydrogen bonds, and the distances of the hydrogen bonds are indicated. **(c)** Detailed depiction of the hydrogen bond formed at the interface of the NTD and CTD of Cry78Aa. The information in the table is completely in accordance with the contents shown in diagram (b). **(d)** Close-up view of the residues that participate in the van der Waals interaction of the NTD and CTD of Cry78Aa. **(e)** Categorization of the residues shown in diagram

(d). **(f)** Coexpression of the NTD and CTD of Cry78Aa in *E.coli*. Both the N-His and C-His tagged CTD of Cry78Aa can pull down its untagged NTD in nickel affinity chromatography. Corresponding bands of the NTD or CTD are indicated by upward or downward arrows. Abbreviations: P, pellets; S, supernatant; F, flow through; W, wash; E, elution; R, resin.

Supplementary Figure 5. Cry78Aa is prone to oligomerize in solution.

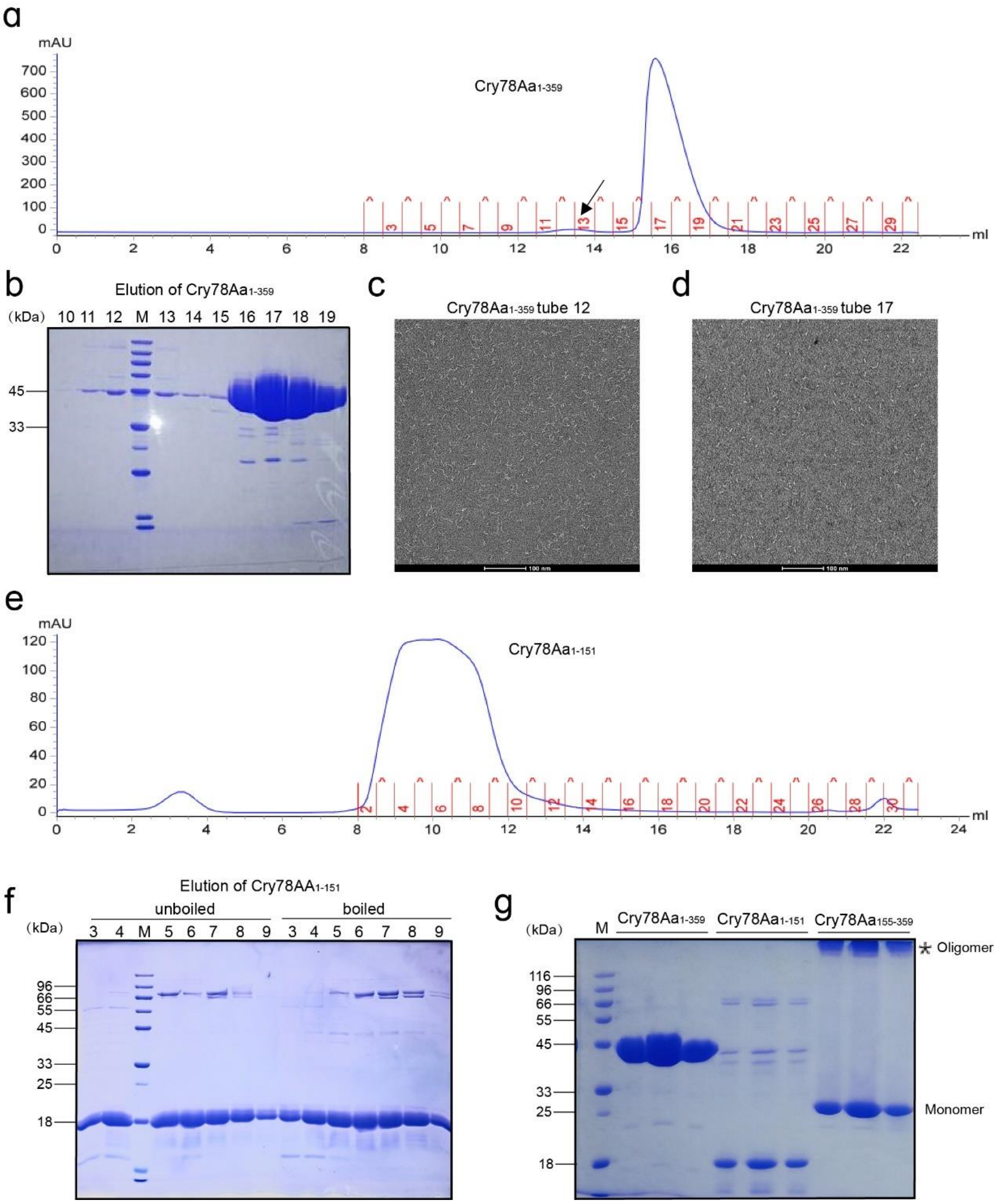

(a) Gel filtration profile of full-length Cry78Aa in 25 mM Tris-HCl, pH 8.0 and 150 mM NaCl. Most of the full-length Cry78Aa protein eluted at approximately 16 ml as a monomer, while a broad elution peak, which may represent oligomeric Cry78Aa, also emerged at approximately 13.5 ml (indicated by the black downward arrow). The numbers and vertical line in red indicate the sequence of samples collected. **(b)** SDS–PAGE detection of the elution peaks collected in gel filtration shown in diagram (a). The numbers above correspond to the tubes collected. The upward arrow indicates oligomeric Cry78Aa, which has been verified by mass spectrometry. **(c)** Negative staining image of the 12th collection of Cry78Aa$_{1\text{-}359}$ in gel filtration. The protein (0.1

mg ml^{-1}) was incubated with uranyl acetate before being subjected to transmission electron microscopy (TEM). **(d)** Negative staining image of the 17th collection of Cry78Aa$_{1-359}$ in gel filtration. The sample treatment procedure is the same as that in (d). **(e)** Gel filtration profile of Cry78Aa$_{1-151}$, which represents the NTD, in 25 mM Tris-HCl, pH 8.0 and 150 mM NaCl. The majority of Cry78Aa$_{1-151}$ eluted at approximately 10 ml, implying the formation of various types of oligomers. The numbers and vertical line in red indicate the sequence of samples collected. **(f)** SDS–PAGE detection of the elution peaks of Cry78Aa$_{1-151}$ exhibited in diagram (e). Samples were boiled or not before loading into the lanes. **(g)** SDS–PAGE detection of the purified full length, NTD or CTD of Cry78Aa. The CTD of Cry78Aa exhibits a strong tendency to oligomerize under mild denature condition (0.1% SDS). The oligomer state of the CTD (indicated by the asterisk) constitutes approximately 30% of the total amount of sample loaded.

Supplementary Figure 6. The trefoil domain of Cry78Aa may bind other sugars comprising β-galactoside bonds.

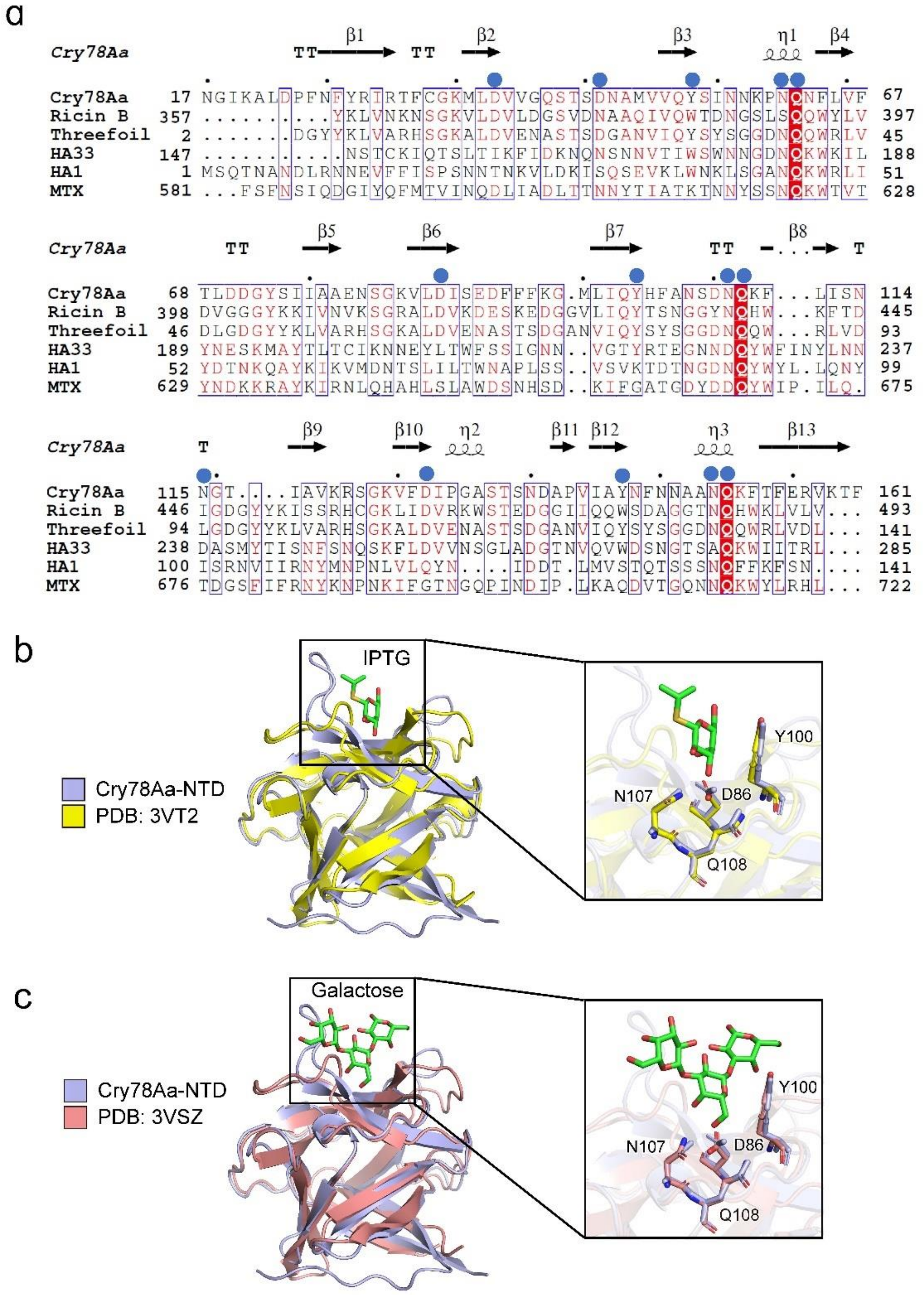

(a) Sequence alignment of the NTD of Cry78Aa and several homologous trefoil domains. Secondary structural units of Cry78Aa are shown above. The numbers flanking the sequence specify the start and ending residues. Conserved residues are marked with a red background. Blue solid circles above indicate the residues taht were mutated for the insecticidal activity test in Fig. 4e. **(b)** Structural alignment of the trefoil

domain of Cry78Aa and the IPTG-bound ricin B lectin domain (PDB: 3VT2). Ribbon diagrams of Cry78Aa and ricin B are shown in light blue and yellow, respectively. The IPTG molecule is shown as a stick. The smaller frame in the left diagram marks the IPTG binding pocket of ricin B, while the larger frame in the right diagram shows the close-up view. The residues of Cry78Aa and ricin B that may interact with IPTG are labeled and shown as sticks (color representations: carbon, light blue for Cry78Aa and yellow for ricin B; nitrogen, blue; oxygen, red; sulfur, ginger), and their orientations are nearly identical. **(c)** Structural alignment of the trefoil domain of Cry78Aa and the multiple galactose-binding ricin B lectin domain (PDB: 3VSZ). The ribbon of ricin B and the galactose molecules (shown as sticks) are colored in deep salmon. Other representations are the same as in diagram (b).

Supplementary Figure 7. Determination of Cry78Aa and sugar binding by isothermal titration calorimetry.

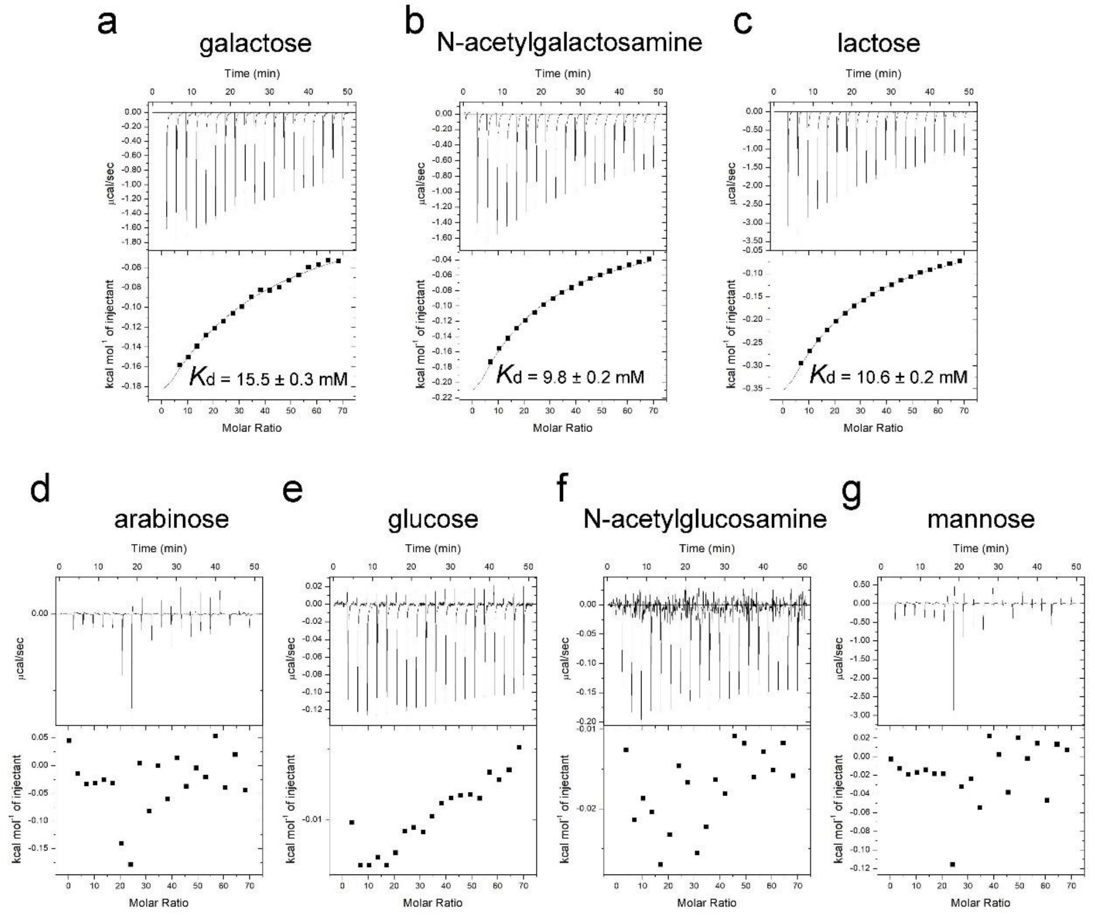

50 mM galactose (a), N-acetylgalactosamine (b), lactose (c), arabinose (d), glucose (e), N-acetylglucosamine (f), and mannose (g) were titrated into 150 µM full-length Cry78Aa. The dissociation constants (K_d) of galactose binding with Cry78Aa is approximately 15.5 mM. N-acetylgalactosamine and lactose exhibited similar binding affinity to Cry78Aa compared with that of galactose. Other sugars showed no detectable interaction with Cry78Aa under the same conditions. Each experiment was repeated twice, and only one result is shown here. The K_d value represents an average of two independent experiments and the error represents the mean deviation.

Supplementary Table 1. Dali search results of the Cry78Aa$_{13-359}$

Chain	Z-score	RMSD (Å)	Residue number	Identity (%)	Description
5FOY-B	31.3	2.5	421	22	De novo structure of the binary mosquito larvicide BinAB
4JP0-A	29.8	2.7	378	22	Crystal structure of Cry35Ab1
2VSE-A	20.2	14.4	823	14	Structure and mode of action of a mosquitocidal holotoxin
3VSF-C	19.7	1.9	482	26	Crystal structure of 1,3Gal43A, an exo-beta-1,3-Galactanase
4ION-A	18.1	1.7	140	21	Macrolepiota procera ricin B-like lectin (MPL)
2IHO-A	17.6	8.3	292	14	A lectin in complex with the trisaccharide Gal(1,3)Gal(1,4)GlcNAc
4G9M-B	17.5	1.7	143	20	Crystal structure of the Rhizoctonia solani agglutinin
2X2S-B	17.3	1.9	152	20	Crystal structure of Sclerotinia sclerotiorum agglutinin SSA
1UPS-A	17.3	1.9	402	17	GlcNAc[alpha]1-4Gal releasing endo-[beta]-galactosidase
1ISV-A	17.3	2.3	436	25	Crystal structure of xylanase complexed with xylose
4LO0-C	16.9	1.7	144	15	Structure of a botulinum neurotoxin complex HA17-HA33
1JLX-A	16.9	1.9	299	13	Structure of benzyl T-antigen disaccharide bound to agglutinin
2VLC-A	16.8	2.2	518	17	Natural ribosome inactivating protein cinnamomin (Isoform III)
3A21-A	16.7	2.4	614	21	Glycoside hydrolase member beta-L-Arabinopyranosidase
6ELY-B	16.7	2.0	264	18	Lectin I in complex with 4-N-Furfurylcytosine
3A07-B	16.1	1.9	118	22	A microbial lectin actinohivin
3WMU-B	16.1	1.8	153	21	A galactose-binding lectin from mussel
3NBC-A	15.9	1.9	148	20	Clitocybe nebularis ricin B-like lectin in complex with lactose
1VC1-A	15.8	2.7	432	21	Hemolytic Lectin CEL-III from Cucumaria echinata
1SR4-A	15.7	2.4	167	15	The Haemophilus ducreyi cytolethal distending toxin
4OWL-D	15.5	2.2	137	14	Hemolysin/Cytolysin Beta-Trefoil Lectin bound with N-Acetyl-D-Lactosamine
4PC4-C	15.4	2.5	240	12	Bombyx mori lipoprotein 6
1W3A-A	15.2	12.7	312	14	Hemolytic pore-forming lectin in complex with sugars
2RST-A	15.0	2.1	132	23	NMR structure of R-type lectin bound with lactose
5AHA-A	14.9	2.0	153	16	Tyrosinase-associated lectin-like protein (MtaL)
3N0K-A	14.9	2.5	150	17	A serine protease inhibitor Cospin (PIC1)
1WD3-A	14.8	2.3	482	9	A family 54 α-L-arabinofuranosidase bound with arabino
3AKI-A	14.7	2.0	448	9	exo-1,5-α-L-arabinofuranosidase complexed with α-L-arabinofuranosyl azido
4A7Z-A	14.6	2.4	881	13	Aldos-2-ulose dehydratase bound with ascopyrone M
2LIE-A	14.2	2.3	153	11	NMR structure of the lectin CCL2
3ZX7-A	14.1	2.1	296	15	Complex of lysenin with phosphocholine
3MAL-A	14.1	3.3	181	6	Arabidopsis stromal-derived Factor2 (SDF2)
1G82-A	14.1	3.3	157	13	Fibroblast growth factor 9
6IWQ-A	14.0	2.5	546	18	Crystal structure of GalNAc-T7 with Mn^{2+}
5XTS-A	13.9	8.1	460	12	CysR-CTLD3 fragment of human mannose receptor

2YUG-A	13.8	2.4	155	13	Solution structure of mouse FRG1 protein
4JKQ-A	13.7	7.9	499	12	N-terminal region of the human ryanodine receptor 2
5VI4-A	13.5	2.2	146	7	Interleukin-33
5AK1-A	13.1	2.0	842	18	Carbohydrate binding family 6
5BOW-A	13.1	2.8	151	14	Interleukin-38
1XEZ-A	13.0	4.1	663	9	Cytolysin pro-toxin bound with octylglucoside
3LTQ-A	12.5	2.6	168	10	Structure of interleukin-1β
5HN1-A	12.3	2.8	157	12	Crystal structure of interleukin-37
5HPZ-A	12.2	2.8	175	12	Type II water soluble chlorophyll binding proteins
3H6R-A	12.0	2.0	152	15	Clitocypin, a beta-trefoil cysteine protease inhibitor
3P6I-A	12.0	1.9	127	14	A de novo designed symmetric beta-trefoil architecture
2KLL-A	11.9	2.2	161	8	Solution structure of human interleukin-33
6MU2-A	11.8	16.5	2170	9	Inositol-1,4,5-trisphosphate receptors 1
1J0S-A	11.8	2.8	157	9	Solution structure of the human interleukin-18
3IIR-A	11.8	2.7	190	9	A miraculin-like protein with trypsin inhibitory activity

Supplementary Table 2. Dali search results of the Cry78Aa$_{13\text{-}155}$

Chain	Z-score	RMSD (Å)	Residue number	Identity (%)	Description
5B2H-A	22	1.4	283	17	HA-33
3AH1-B	21.6	1.5	288	12	HA1 Subcomponent of Botulinum Type C Toxin
3VSF-C	20.7	1.8	482	26	1,3Gal43A
2VSE-A	20.6	1.9	823	15	Mosquitocidal Toxin
3PG0-A	20.5	1.7	140	35	Threefoil
4LO0-A	20.2	1.8	286	21	HA-33
3WIN-A	19.5	1.6	286	25	HA3
4ION-A	18.9	1.7	140	20	Macrolepiota Procera Ricin B-Like Lectin (MPL)
4Z8S-B	18.9	1.9	261	16	Rrna N-Glycosidase
4G9M-B	18.8	1.5	143	21	Agglutinin
3CA6-A	18.8	1.6	258	15	Agglutinin II
1HWM-B	18.7	1.9	264	16	Ebulin
2IHO-A	18.5	1.8	292	16	MOA
1ABR-B	18.3	1.8	267	15	Abrin-A
1ISV-A	18.2	2.3	436	25	Endo-1,4-β-D-Xylanase
1UPS-A	18.2	1.9	402	16	Glcnac-α-1,4-Gal-Releasing Endo-β-Galactosidase
2X2S-B	18.2	2	152	20	Agglutinin
5XG5-A	18.2	1.4	145	24	Mitsuba-1
1RZO-B	18.2	1.7	262	19	Agglutinin
6IFB-B	18.1	1.6	134	20	Lectin
4JP0-A	18.1	2.2	378	22	Cry35Ab1
3PHZ-A	18.1	2	278	19	PSL
2ZR1-B	17.9	1.7	263	16	Agglutinin-1 Chain A
6ELY-B	17.9	1.7	264	18	Mistletoe Lectin I
5FOY-A	17.8	1.8	367	16	BinA
1GGP-B	17.8	1.9	254	18	TKL-1
3A21-A	17.7	2	614	22	Putative Secreted α-Galactosidase
4HR6-C	17.7	1.9	264	13	SGSL
4LO0-C	17.6	1.6	144	16	HA-33
1KNM-A	17.6	2	129	23	Endo-1,4-β-Xylanase A;
3Q7Y-A	17.6	1.7	124	13	De Novo Designed β-Trefoil
1JLX-A	17.5	1.8	299	13	Agglutinin

2VLC-A	17.4	1.9	518	18	Natural Cinnamomin (Isoform III)
1CE7-B	17.4	2.1	255	14	Mistletoe Lectin I
3A07-B	17.3	1.8	118	22	Actinohivin
2E4M-C	17.3	1.8	143	14	Hemagglutinin Subcomponent Complex (HA-33/HA-17)
4QKR-A	17.2	1.7	125	13	6xTyr/PV2
3O4C-A	17.1	1.7	124	14	De Novo Designed β-Trefoil
5DUY-A	17	1.5	150	22	CGL
4IY8-A	16.9	2.3	239	11	Bmlp3-P21
1VCL-A	16.8	2.1	432	24	Hemolytic Lectin CEL-III
2AAI-B	16.8	2	262	19	Ricin (A Chain)
3WMU-B	16.7	1.6	153	21	MytiLec
4I4Q-A	16.5	2.2	146	16	Bel-β Trefoil
6EUF-A	16.4	1.9	472	14	β-Glucanase
4OW4-A	16.4	1.7	123	13	β-Terfoil (Designed)
1W3A-A	16.3	2.5	312	16	Hemolytic Lectin LSLA
3NBC-A	16.3	2	148	21	CNL
1SR4-A	16.2	2.5	167	16	CdtA
4PC4-C	16.2	2.5	240	10	Bmlp6
4I4Y-B	16.2	2.3	149	15	Bel β-Trefoil
3KMV-E	16.2	1.9	140	11	α-L-Arabinofuranosidase B
4OWL-D	15.9	2.3	137	13	Cytolysin
4CJM-A	15.9	1.9	129	14	FGF18
3N0K-A	15.8	2.4	150	17	Cospin (PIC1)
5FOY-B	15.7	2.2	421	18	BinB
6S24-A	15.5	2	537	19	GalNAc-T3
5ZXE-A	15.5	2	130	18	FGFs
2RST-A	15.4	2	132	22	EW29(Ch; C-half)
1QQL-A	15.4	2	131	13	FGF 7/1 Chimera
6SXT-A	15.3	2.1	483	9	α-L-Arabinofuranosidase B
3AKI-A	15.3	1.8	448	9	Putative Secreted α L-Arabinofuranosid
6PXU-B	15.3	2.1	532	18	Polypeptide N-Acetylgalactosaminyltransfer
1G82-A	15.2	2.3	157	14	FGF 9
5EHA-A	15.2	2.4	153	16	MtaL
1QQK-A	15.2	2	129	13	FGF 7
5GUG-A	15.2	2.2	1721	10	IP_3 R

4L4I-A	15.2	1.9	473	11	RyR2
4A7Z-A	15.1	2.3	881	12	AUDH
1NUN-A	15.1	1.9	139	17	FGF 10
4JQ0-A	15.1	2.1	141	10	FGF 12
3MAL-A	15.1	2.5	181	6	SDF2-Like Protein
1T9F-A	15	2.2	178	7	Protein 1d10
2FDB-M	15	1.9	149	14	FGF 8 Isoform B
1IJT-A	15	1.9	128	12	FGF 4
6H0B-B	14.9	2.2	521	19	GalNAc-T4
1RY7-A	14.9	1.9	151	12	FGF 1
4JKQ-A	14.9	2.1	499	10	RyR 2
6IWQ-A	14.8	2.4	546	18	GalNAc-T7
3ZX7-A	14.8	2.4	296	14	Lysenin
3HBW-A	14.8	2.2	149	12	FGF 13
2LIE-A	14.7	1.9	153	12	CCL2 Lectin
6P25-A	14.5	2.3	731	9	PMT1
3F1R-A	14.5	2.1	157	13	FGF 20
1BLA-A	14.5	2.1	155	12	FGF2
5Y9V-A	14.5	2.3	177	9	RyR 1
1DQG-A	14.4	2.1	134	15	MR
1XHB-A	14.4	2.4	447	18	ppGaNTase-T1
4I7I-A	14.4	2.2	485	9	RyR 1
2MC2-A	14.3	2.4	206	11	RyR 2
1XEZ-A	14.2	2.4	663	10	Hemolysin
6INN-A	14.2	2.1	598	16	MRC1
2YUG-A	14.2	2.3	155	13	Mouse FRG1
6E4Q-A	14.1	2.4	505	18	PANT9
5AK1-A	13.9	1.9	842	17	CtXyl5A
6P25-B	13.9	2.5	690	12	PMT1
6IOE-B	13.8	2.2	446	16	MRC1
5VI4-A	13.8	2.2	146	6	IL33
5BOW-A	13.7	2.4	151	14	IL1F10
3QR5-B	13.7	2	154	8	RyR2
2D7R-A	13.5	2.5	537	16	pp-GalNAc-T10
6UQK-A	13.5	2.4	2053	13	ITPR3
4D0T-A	13.4	2.5	496	15	GalNAc-T2

2MIB-A	13.3	2.6	149	15	IL1 β
4GAF-A	13.1	2.4	152	15	EBI-005
3V0A-B	12.9	2.9	1150	21	BoNT/A
3LTQ-A	12.8	2.5	168	11	IL1 β
3OBR-A	12.8	2.6	418	11	Bot D
5MVZ-V	12.8	2.6	144	10	Fab 4AB007 H-Chain
5HPZ-A	12.7	2.9	175	12	WSCP1
3R4S-A	12.7	2.5	424	10	Bot C1
2P23-A	12.6	2.4	136	10	FGF 19
5HN1-A	12.6	3	157	13	IL37
4IZE-A	12.6	2.6	152	12	IL36 γ
3H6R-A	12.5	1.9	152	16	CLT2
3P6I-A	12.5	1.9	127	13	De Novo Designed β-Trefoil
4P0J-A	12.5	2.7	147	13	IL36G
4X37-A	12.5	2.6	159	7	IL1 β
3IIR-A	12.4	2.8	190	8	Trypsin Inhibitor
3AZV-A	12.4	2.7	418	8	D/C Mosaic BoNT
2KLL-A	12.3	2.2	161	8	IL-33
4IHZ-A	12.3	2.4	164	10	CrataBL
1J0S-A	12.2	2.6	157	9	IL-18
2DRE-C	12.2	2.7	177	13	WSCP1
3S8K-A	12.1	2.8	183	14	Latex Serine Proteinase Inhibitor
4AN6-A	12.1	2.9	172	13	Trypsin Inhibitor
6MU2-A	12	2.7	2170	9	InsP3R
1MD6-A	11.9	2.9	154	14	IL-1F5
5YH4-A	11.9	2.9	179	13	Mirauclin-Like Protein
2GO2-A	11.9	2.5	163	9	BbKI
5YCZ-A	11.9	3	178	15	Trypsin/Chymotrypsin Inhibitor
2GZB-A	11.8	3	164	12	BbCI
6HOX-A	11.8	2.8	421	11	PMP1
5DVH-A	11.8	2.9	185	16	PCPI-3
5FNW-A	11.8	2.9	185	15	Bi-functional Kunitz-Type STI
4J2K-A	11.8	2.8	168	16	Trypsin Inhibitor
1WBA-A	11.7	2.7	171	17	Winged Bean Albumin 1
5W21-B	11.7	2	182	15	Klotho
3H6Q-A	11.7	2.7	168	11	Macrocypin 1A

3VUO-A	11.6	3	1151	16	Ntnha
3TC2-A	11.6	2.7	181	13	PSPI
1R8N-A	11.6	2.8	185	10	Kunitz Trypsin Inhibitor
6ES1-A	11.6	2.8	420	13	BoNT/A2
6JBP-B	11.6	2.9	179	15	MP-4
4TLV-A	11.5	2.8	582	11	ADP-Ribosylating Toxin Cards
5XOZ-B	11.5	2.9	179	10	Trypsin Protein Inhibitor 2
4ZKT-B	11.5	2.7	1114	12	Bontoxilysin A
1AVA-C	11.4	3.1	181	10	AMY2
5DZU-A	11.4	2.7	187	14	PDI
1TIE-A	11.4	2.5	166	9	Erythrina Trypsin Inhibitor
1WBC-A	11.3	2.7	183	8	Chymotrypsin Inhibitor (WCI)
2QN4-A	11.2	2.8	159	11	RASI
2VXR-A	10.9	3	428	11	BoNT/G
3V0A-A	10.9	2.8	1280	12	BoNT/A
2KKI-A	10.8	2.6	151	7	IL-1 α
3E8L-C	10.8	2.6	176	9	Serine Proteinase Inhibitor A
3FFZ-A	10.8	2.8	1246	9	BoNT/E
3MPP-G	10.8	3	403	12	BoNT/G
6O1F-I	10.7	2.9	177	10	Tryptase α/β-1
1FMM-S	10.7	2.8	132	16	FGF1
6KV2-B	10.6	2.7	191	13	Trypsin Inhibitor 1
3RSJ-A	10.6	2.8	406	8	BoNT/F
5MK8-A	10.6	2.8	421	14	BoNTs H_C/FA
5J8V-A	10.5	2.2	3398	7	RyR 1
1HCD-A	10.5	2.8	118	13	Hisactophilin
6B0T-A	10.5	2	487	9	Fascin
1SR4-C	10.4	2.4	154	8	CDT A
5VID-E	10.4	2.7	405	11	BoNT/B
5DSS-B	10.4	2.8	185	12	Mp-4
2NP0-A	10.4	2.8	1289	10	BoNT/B
5N0C-A	10.2	2.8	1293	10	Tetanus Toxin
6HPI-A	9.7	3.2	158	11	IL-36 α
3JAV-A	9.6	2.7	868	10	IP3R1
1IRP-A	9.5	3	153	15	IRAP
5BXP-A	9.5	2.5	636	19	LNBase

6PY8-E	9.5	2.3	431	13	NRARP
5E24-E	9.5	1.8	424	13	Maltose-Binding Periplasmic Protein
3ILA-I	9	1.4	109	8	RyR 1
2FO1-A	8.6	2.4	439	11	CSL-Notch-Mastermind
6INU-B	8.5	1.9	430	20	MRC1
5E4L-B	8.2	2.9	386	12	uPARAP
4D11-C	7.2	2.6	436	13	GalNAc-T2
5EW6-A	7	2.4	442	12	uPARAP
1R8O-A	3.4	2.9	96	17	Kunitz Trypsin Inhibitor
1R8O-B	3.3	2.9	71	7	Kunitz Trypsin Inhibitor
3OL0-A	3.2	1.7	43	13	De Novo Designed Monomer Trefoil-Foldsub-domain
5HBB-C	3.1	3.3	267	13	Cell Surface Protein SpaA
1ZQ1-A	2.9	3	438	13	Glutamyl-tRNA(Gln) Amidotransferase SubunitD
4UZG-A	2.8	3.2	277	9	Surface Protein SPB1
3HFO-B	2.7	3.6	66	10	Ssr3341 Protein
4ZU9-A	2.7	2.8	583	14	Elongation Factor SeIB
2CAY-A	2.7	3	132	10	Vps 36
3FB9-A	2.5	2.7	89	7	Uncharacterized Protein
6NDL-A	2.5	2.6	323	15	*Sa* BPL
5Z0Z-B	2.5	3	450	2	Pilus Assembly Protein
1Y96-A	2.5	3.6	86	11	GEMIN6
2EC1-A	2.4	2.7	118	13	NUP50
5N8O-A	2.4	3	1432	7	DNA Helicase I
5EAN-A	2.3	2.8	1051	11	Dna2 nuclease-helicase
2Y1V-A	2.3	3.3	604	11	RrgB Pilus protein
6GWK-B	2.3	3.8	77	15	RNA Binding Protein Hfq;
4XI6-A	2.3	3.9	363	4	the MZM-REP domains of Mind bomb 1
2J05-A	2.2	2.9	64	19	RasGAP SH3 domain
6AM0-D	2.2	3.1	65	10	Klla0f23980p
2HTH-B	2.1	3.7	129	5	Ubiquitin
3OAN-A	2.1	2.6	116	12	ABR034Wp
3FC3-A	2.1	3	189	7	Restriction Endonuclease Hpy99I
6QGI-A	2.1	3.5	498	15	Vp5
4ZKE-A	2	3	473	8	Ski7
6JCH-A	2	3.9	327	11	Pilus Assembly Protein
2HEQ-A	2	3.6	84	10	Yorp Protein

Supplementary Table 3. Dali search results of the Cry78Aa$_{155-359}$

Chain	Z-score	RMSD (Å)	Residue number	Identity (%)	Description
5FOY-A	24.6	1.9	367	26	BinA
4JP0-A	21.5	2.2	378	23	Cry35Ab1
5FOY-B	17.9	2.7	421	24	BinB
4MJT-A	5.2	4.1	236	10	Monalysin
6LH8-A	4.2	3.9	152	10	Aerolysin-like protein
2D42-A	4.2	4.8	249	9	Non-toxic crystal protein
3ZX7-A	4.0	3.7	296	10	Lysenin
1W3A-A	4.0	4.9	312	5	Hemolytic lectin LSLA
4ZNO-A	3.8	4.9	318	10	Parasporin
1VCL-A	3.6	3.6	432	9	Hemolytic lectin CEL-III
5ZU4-B	3.6	4.2	314	17	Natterin-like protein
2ZTB-B	3.5	4.3	248	10	Parasporin-2 (Cry46Aa1)
4J0X-B	3.3	6.0	366	6	Rrp9
3ZJX-A	3.1	4.2	277	6	Epsilon-toxin
5CVO-D	3.1	3.5	612	7	WDR 48
4O9D-B	3.0	6.5	392	3	Rik1-associated factor 1
4RHZ-A	2.8	4.2	259	5	Cry23Aa1
3NO2-A	2.8	5.5	274	5	Uncharacterized protein
3LP9-A	2.6	4.1	227	2	LS-24
3TEK-A	2.5	3.9	139	4	ThermoDBP-single stranded DNA binding protein
6BAQ-F	2.5	4.2	211	5	BPIFA1
6IQT-A	2.5	4.7	135	1	Cag pathogenicity island protein (Cag10)
4MKQ-A	2.5	4.4	169	7	Monalysin
5ZBT-A	2.3	2.6	212	2	ERGIC-53-like protein
3R90-J	2.3	4.0	188	15	Malignant T cell-amplified sequence 1
6JP6-A	2.3	8.1	998	4	TRM734
5YX4-A	2.3	3.3	232	10	CHI1
6IF4-B	2.2	4.4	61	7	TbTudor
3KOG-A	2.2	5.5	226	8	Putative pore-forming toxin
1SS4-A	2.2	2.7	149	4	Glyoxalase family protein
5UCR-A	2.2	3.7	271	12	Pantothenate synthetase
6W2E-J	2.1	3.7	1305	10	Major capsid protein
1PRE-B	2.1	6.9	451	3	Proaerolysin
