# Supplementary material for: The crystal structure of Cry78Aa from Bacillus thuringiensis provides insights into its insecticidal activity
Source: Commun Biol. 2022 Aug 9;5:801. doi: 10.1038/s42003-022-03754-6 (PMC9363482; doi:10.1038/s42003-022-03754-6)
Supplement: Supplementary file 3 — Description of Additional Supplementary Files [file 42003_2022_3754_MOESM3_ESM.pdf]

Description of Additional Supplementary Files

File name: Supplementary Data 1
Description: The source data behind the graphs presented in the main figures of the paper.

File name: Supplementary Data 2
Description: Uncropped western gel results which was used in Figure 2e.
