# Supplementary material for: The crystal structure of Cry78Aa from Bacillus thuringiensis provides insights into its insecticidal activity
Source: Commun Biol. 2022 Aug 9;5:801. doi: 10.1038/s42003-022-03754-6 (PMC9363482; doi:10.1038/s42003-022-03754-6)
Supplement: Supplementary file 5 — Supplementary Data 2 [file 42003_2022_3754_MOESM5_ESM.pdf]

Supplementary Data 2. The BBMV of *L. striatellus* promoted Cry78Aa oligomer formation. An uncropped western results of Figure 2e in the main text.

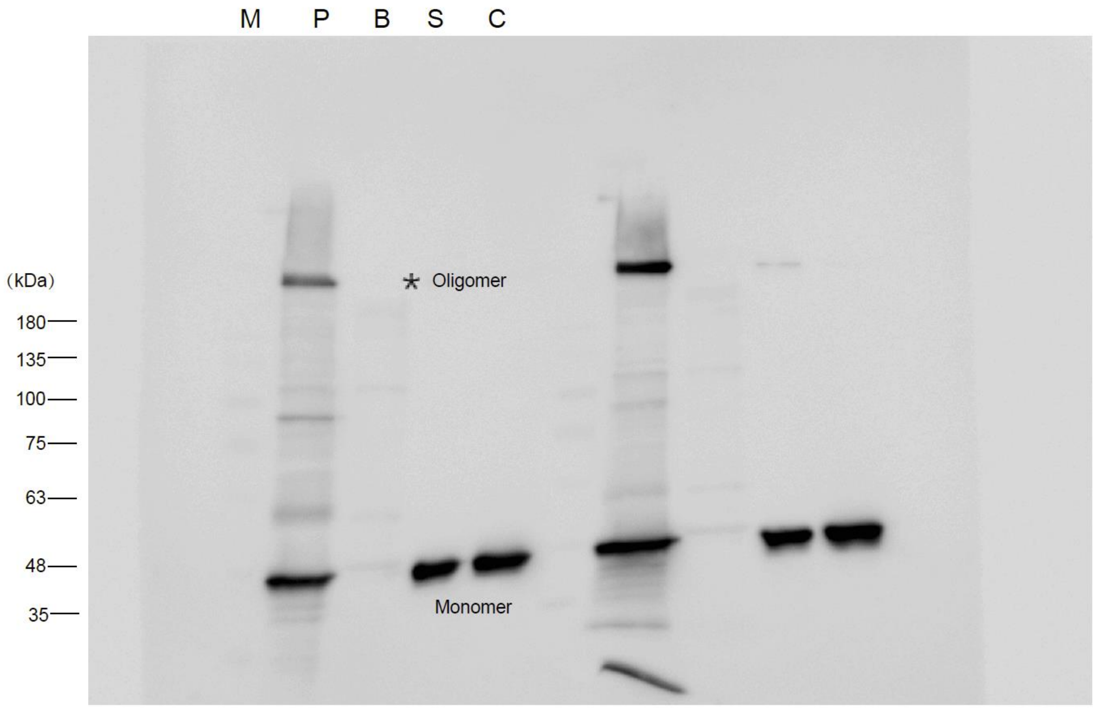

Lane M: Molecular weight marker PR1910 (Solarbio, Beijing).
Lane P: Pellet obtained after incubation of the Cry78Aa protein with BBMVs of L. striatellus.
Lane B: BBMVs incubated without Cry78Aa.
Lane S: Supernatant obtained after incubation of Cry78Aa with BBMVs diluted 50 times.
Lane C: Cry78Aa protein incubated without BBMVs, containing 60 ng of Cry78Aa protein.
