# Supplementary material for: The crystal structure of Cry78Aa from Bacillus thuringiensis provides insights into its insecticidal activity
Source: Commun Biol. 2022 Aug 9;5:801. doi: 10.1038/s42003-022-03754-6 (PMC9363482; doi:10.1038/s42003-022-03754-6)
Supplement: Supplementary file 6 — Reporting Summary [file 42003_2022_3754_MOESM6_ESM.pdf]

nature portfolio

Corresponding author(s): DBPR COMMSBIO-21-1677-A

Last updated by author(s): Jun 23, 2022

Reporting Summary

Nature Portfolio wishes to improve the reproducibility of the work that we publish. This form provides structure for consistency and transparency in reporting. For further information on Nature Portfolio policies, see our Editorial Policies and the Editorial Policy Checklist.

Statistics

For all statistical analyses, confirm that the following items are present in the figure legend, table legend, main text, or Methods section.

n/a	Confirmed	
☐	☒	The exact sample size (n) for each experimental group/condition, given as a discrete number and unit of measurement
☐	☒	A statement on whether measurements were taken from distinct samples or whether the same sample was measured repeatedly
☐	☒	The statistical test(s) used AND whether they are one- or two-sided<br>*Only common tests should be described solely by name; describe more complex techniques in the Methods section.*
☐	☒	A description of all covariates tested
☐	☒	A description of any assumptions or corrections, such as tests of normality and adjustment for multiple comparisons
☐	☒	A full description of the statistical parameters including central tendency (e.g. means) or other basic estimates (e.g. regression coefficient) AND variation (e.g. standard deviation) or associated estimates of uncertainty (e.g. confidence intervals)
☐	☒	For null hypothesis testing, the test statistic (e.g. F, t, r) with confidence intervals, effect sizes, degrees of freedom and P value noted<br>*Give P values as exact values whenever suitable.*
☐	☒	For Bayesian analysis, information on the choice of priors and Markov chain Monte Carlo settings
☐	☒	For hierarchical and complex designs, identification of the appropriate level for tests and full reporting of outcomes
☐	☒	Estimates of effect sizes (e.g. Cohen's d, Pearson's r), indicating how they were calculated

Our web collection on statistics for biologists contains articles on many of the points above.

Software and code

Policy information about availability of computer code

Data collection	HKL3000, HKL2000, the Protein Data Bank (PDB), the pesticide protein database of the Bacterial Pesticide Protein Resource Center (https://www.bpprc.org/)
Data analysis	PHENIX, COOT,PyMOL(http://www.pymol.org), Origin 8.0, MEGA-X (10.0.2), SPSS 22.0, the website of Interactive Tree of Life (https://itol.embl.de/).

For manuscripts utilizing custom algorithms or software that are central to the research but not yet described in published literature, software must be made available to editors and reviewers. We strongly encourage code deposition in a community repository (e.g. GitHub). See the Nature Portfolio guidelines for submitting code & software for further information.

Data

Policy information about availability of data

All manuscripts must include a data availability statement. This statement should provide the following information, where applicable:

- Accession codes, unique identifiers, or web links for publicly available datasets
- A description of any restrictions on data availability
- For clinical datasets or third party data, please ensure that the statement adheres to our policy

The datasets generated during and/or analysed during the current study are available from the corresponding author on reasonable request.

Field-specific reporting

Please select the one below that is the best fit for your research. If you are not sure, read the appropriate sections before making your selection.

☒ Life sciences ☐ Behavioural & social sciences ☐ Ecological, evolutionary & environmental sciences

For a reference copy of the document with all sections, see nature.com/documents/nr-reporting-summary-flat.pdf

Life sciences study design

All studies must disclose on these points even when the disclosure is negative.

Sample size	Sample sizes were chosen based on preliminary data demonstrating statistically significant differences for each specific assay.
Data exclusions	Data exclusions were not pre-established. Data were excluded where the investigator noted an technical error that may have affected results. Where errors were determined (based on results e.g. whether control samples performed as expected), they were excluded from analyses.
Replication	All experiments were performed with at least three technical replicates on more than one occasion to ensure reproducibility across experiments.
Randomization	Randomization and covariates were not relevant to our study design as we investigated single factors within each study.
Blinding	Bioactivity experiments were not blinded as only 1 investigator conducted each study.

Reporting for specific materials, systems and methods

We require information from authors about some types of materials, experimental systems and methods used in many studies. Here, indicate whether each material, system or method listed is relevant to your study. If you are not sure if a list item applies to your research, read the appropriate section before selecting a response.

Materials & experimental systems

n/a	Involved in the study	
☐	☒	Antibodies
☒	☐	Eukaryotic cell lines
☒	☐	Palaeontology and archaeology
☐	☒	Animals and other organisms
☒	☐	Human research participants
☒	☐	Clinical data
☒	☐	Dual use research of concern

Methods

n/a	Involved in the study	
☒	☐	ChIP-seq
☒	☐	Flow cytometry
☒	☐	MRI-based neuroimaging

Antibodies

Antibodies used	Cry78Aa1 murine monoclonal antibody (Genecreate, Wuhan, China) is suitable for the detection of Cry78Aa1 protein by Western blot or Elisa, Cat. Number:20190717, Lot. Number:20190924. Goat Anti-Mouse IgG/HRP (Solarbio, Beijing, China) is used for enzyme immunoassay, Cat. Number:SE131.
Validation	Cry78Aa1 mouse monoclonal antibody was customized in Genecreate, and it specifically recognizes Cry78Aa1 protein. The optimal dilution ratio for western blot assay was 1:8 000.

Animals and other organisms

Policy information about studies involving animals; ARRIVE guidelines recommended for reporting animal research

Laboratory animals	Laodelphax striatellus was raised with rice seedlings in the artificial climate incubator of our laboratory, and its population was expanded. The third instar nymphs of Laodelphax striatellus were used for insecticidal activity measurement in this study.
Wild animals	This study did not involve wild animals.
Field-collected samples	This study did not involve samples collected from the field.
Ethics oversight	Animal ethics are not involved in this study. In this study, Laodelphax striatellus is an invertebrate, and the method and purpose of the biological test are in line with human ethical standards and international practices.

Note that full information on the approval of the study protocol must also be provided in the manuscript.
